# Supplementary material for: Exploring Parents’ Concerns Regarding Long-Term Support and Living Arrangements for Their Children with Fragile X Syndrome
Source: Genes (Basel). 2022 Sep 15;13(9):1654. doi: 10.3390/genes13091654 (PMC9498767; doi:10.3390/genes13091654)
Supplement: Supplementary file 1 [file genes-13-01654-s001.zip › genes-1886118-supplementary.pdf]

Project Questionnaire
Title: Living arrangements and long-term supports for adults with FXS.

Eligibility Criteria
-Only family members are eligible to complete this questionnaire
-The term 'person' is used to refer to the adult individual with FXS who is the subject of the survey
-If you care for more than one individual with FXS please complete the survey in regards to the eldest individual

Eligibility Questions

1. Does the individual with FXS have a full mutation of the *FMR1* gene?
    - Yes
    - No
    - Unsure

2. What is your relationship to the person with FXS?
    - Mother
    - Father
    - Other family member

3. How many individuals diagnosed with FXS do you care for?
    - 1
    - 2
    - 3 or more

A) Demographics/Caregiver Information

1. Which race/ethnicity best describes you? (Please choose only one)
    - White
    - Hispanic or Latino
    - Black or African American
    - Native American or American Indian
    - Asian or Pacific Islander
    - Prefer not to answer
    - Other

2. Which region of the U.S. do you currently reside?

- Northeast (ME, NH, VT, MA, RI, CT, NY, NJ, PA)
- South (DE, MD, VA, WV, KY, NC, SC, TN, GA, FL, AL, MS, AR, LA, OK, TX)
- Midwest (OH, MI, IN, IL,WI, MN, IA, MO, ND, SD, NE, KS)
- West (MT, ID, WY, CO, NM, AZ, UT, NV, CA, OR, WA, AK, HI)
- Other: [fill in the blank]

3. What is the highest degree or level of school you have completed?
    - Less than high school
    - Some high school
    - High school degree or equivalent (GED)
    - Technical school/some college/associate's degree
    - College degree (bachelor's)
    - Post graduate degree (master's/doctorate)
    - Prefer not to answer

4. What is your average annual household income?
    - Less than $50,000
    - $50-100,000
    - $100-150,000
    - $150-200,000
    - More than $200,000
    - Prefer not to answer

B) FXS Individual Characteristics

1. What is the sex of the individual with FXS?
    - Male
    - Female

2. Select the age group the person falls under
    - 15-24 years
    - 25-34 years
    - 35-44 years
    - 45-54 years
    - ≥55 years

3. Does the person have any of the following conditions?
   Matrix: 0 = no, 1 = unsure, 2 = mild, 3 = moderate, 4 = severe
   - Intellectual Developmental Disability (IDD)
   - Autism Spectrum Disorder
   - Attention problems
   - Hyperactivity
   - Constant aggressive behavior
   - Intermittent explosive disorder (sudden episodes of impulsive, aggressive, violent behavior) (Note: select mild if episodes are separated by months, moderate if separated by weeks, and severe if episodes occur weekly)
   - Self-injury
   - Anxiety
   - Depression

4. Does the person have any additional medical issues? (select all that apply)
   - Seizures
   - Gastrointestinal (GERD, acid reflux)
   - Sleep disorders (insomnia, sleep apnea)
   - Hearing loss
   - Vision (strabismus, lazy eye)
   - Cardiac (mitral valve prolapse, aortic root dilated)
   - None
   - Other: [fill in the blank]

5. How would you rate this person's overall health?
   - 1 = very poor
   - 2 = poor
   - 3 = fair
   - 4 = good
   - 5 = excellent

6. Does the person require assistance with the following skills?

Matrix: 0 = independent or does on own, 1 = does with help, 2 = does not do at all

- Making his/her own bed
- Doing household tasks, including picking up around the house, putting things away, light house cleaning, etc
- Doing errands, including shopping in stores
- Doing home repairs, including simple repairs around the house, non-technical in nature; for example, changing light bulbs or repairing a loose screw
- Doing laundry, washing and drying
- Washing/bathing
- Grooming, brushing teeth, combing and/or brushing hair
- Dressing and undressing
- Toileting
- Preparing simple foods requiring no mixing or cooking, including sandwiched, cold cereal, etc.
- Mixing and cooking simple foods, fry eggs, make pancakes, heat food in microwave, etc.
- Preparing complete meal
- Setting and clearing table
- Drinking from a cup
- Eating from a plate
- Washing dishes (including using a dishwasher)
- Banking and managing daily finances, including keeping track of cash, checking account, paying bills, etc. (Note: if he/she can do a portion but not all, check 'does with help')

7. Does the person do any of the following activities? (select all that apply)
    - Volunteer
    - Work, part-time
    - Work, full-time

C) Living Arrangements/Supports

1. Where does the individual with FXS currently reside?
    - Residing with parents or family members [*branch to Q2/Q3*]
    - Host/Group home [*branch Q6-10*]
    - Residential facility [*branch Q6-10, Q12*]

- Independent apartment/house with support (staff may come throughout day or week) [*branch to Q3*]
- Independent living or with a partner [*branch to Q3*]
- Other: [fill in the blank] [*branch to Q3*]

2. How much do the following factors apply to the reason they live at home?

Matrix: 0 = not a reason, 1 = minor, 2 = neutral, 3 = moderate, 4 = major reason

- Parent desire
- Individual with FXS desire
- Limited residential options/currently on waitlist
- Not satisfied with available residential services
- Rejected from residential placement/group home: [*provide reason*]
- Child unable to live independently
- Have not considered other living arrangements

3. What future living arrangement have you considered most for this person? (If same as where they are currently living, select that choice)
    - Live with family members long term [*branch to Q5]*
    - Host/Group home [*branch to Q4*]
    - Residential facility [*branch to Q4]*
    - Independent apartment/house with support [*redirect to Q11, Q13*]
    - Independent living or with a partner [*redirect to Q11, Q13*]
    - Not yet considered/Unsure [*branch to Q5]*
    - Other: [fill in the blank] [*branch to Q4*]

4. Have you made plans or proceeded to enroll them in out-of-home living arrangements?
    - No [*branch to Q5*]
    - Yes [*branch to Q6-10, Q12]*

5. Reason(s) you have not started planning or considered alternative living arrangements: (select all that apply) [*redirect to Q11, Q13*]
    - Lack of information
    - Lack of time
    - No residential services near me

- Not satisfied with living arrangement options
- Confused on where to start
- Overwhelmed with information
- Not a concern yet/still have time
- Other: [fill in the blank]

6. How important were/are the following factors when searching for a residence?

Matrix: 0 = have not considered 1 = not important, 2 = slightly important, 3 = moderately important, 4 = very important, 5 = extremely important

- Location
- Price
- Health services
- Educational services
- Recreational activities
- Staff to resident ratio
- Available activities
- Ability to visit
- Day programs

7. Which of the following factors were/are difficult to satisfy during your search? (select all that apply)
    - Location
    - Price
    - Health services
    - Educational services
    - Recreational activities
    - Staff to resident ratio
    - Available activities
    - Ability to visit
    - Day programs

8. If your child has behavioral issues, did/does it affect finding a residence?
    - My child does not have behavioral issues
    - No, it did not affect it

- Yes, it did affect it: [which behavioral issues and how]

9. How much does the residential facility cost annually?
    - Less than $50,000
    - $50-100,000
    - $100-150,000
    - More than $150,000
    - Prefer not to answer

10. How do you afford/plan to afford this residential service? (select all that apply)
    - Public funding (i.e. Supplemental security income, Medicaid waiver)
    - Special needs trust (ABLE Act)
    - Self-pay by parent/caregiver
    - Insurance
    - Income from person's job
    - Other: [fill in the blank]

11. How would you rate their satisfaction with their currently living arrangement? [*not branched*]
    - Very Dissatisfied: [provide reason]
    - Dissatisfied: [provide reason]
    - Neutral
    - Satisfied: [provide reason]
    - Very satisfied: [provide reason]

12. Does the residential facility provide any of the following health services? If yes, select your satisfaction level with them.
    0 = unsure if provided, 1 = does not provide, 2 = provides, not satisfied, 3 = provides, neutral 4 = provides, satisfied
    - Residential medical care or nursing
    - Physical therapy
    - Speech therapy
    - Behavioral therapy
    - Psychological support staff

- Occupational therapy or direct support professionals
- Dietician/nutritionist

13. What would be the ideal living arrangement for the individual with FXS? [free response] [*not branched*]

D) Future Concerns

1. How concerned are you about the future in the following areas?

Matrix: 1 = not at all concerned, 2 = slightly concerned, 3 = somewhat concerned, 4 = moderately concerned, 5 = extremely concerned

- ○ Who will care for them when I am no longer able to
- ○ Financial support
- ○ The availability of resources
- ○ The quality of resources
- ○ The transition to different long-term supports/living arrangements
- ○ Abuse when I am not here anymore

2. Have you started making plans to address these concerns?
    - ○ Yes: [explain how]
    - ○ No: [provide reason(s)]

3. In the future, how will the person be supported financially? (select all that apply)
    - ○ Public funding (i.e. supplemental security income, Medicaid waivers)
    - ○ Special needs trust (ABLE Act)
    - ○ Self-pay by parent/caregiver
    - ○ Insurance
    - ○ Income from their job
    - ○ Unsure
    - ○ Other: [fill in the blank]

4. Who will oversee their medical decisions when you are no longer able to?
    - ○ Close family member (sibling, aunt, uncle)
    - ○ Friends/other family
    - ○ Residential facility director

- My child will make his/her own decisions
- Have not considered/unsure
- Other: [fill in blank]

5. Who will oversee their financial decisions when you are no longer able to?
    - Close family member (sibling, aunt, uncle)
    - Friends/other family
    - Residential facility director
    - My child will make his/her own decisions
    - Have not considered/unsure
    - Other: [fill in blank]

E) Free Response (Optional)

1. Please share any additional concerns you have regarding future long-term supports/living arrangements for the individual with FXS that were not covered in the questionnaire.

2. If applicable, please share your experiences addressing these concerns.
